# A Review of Cell Adhesion Studies for Biomedical and Biological Applications

**DOI:** 10.3390/ijms160818149

**Published:** 2015-08-05

**Authors:** Amelia Ahmad Khalili, Mohd Ridzuan Ahmad

**Affiliations:** 1 Department of Control and Mechatronic Engineering, Faculty of Electrical Engineering, Universiti Teknologi Malaysia, Johor 81310, Malaysia; E-Mail: amelia.ahmadkhalili@gmail.com; 2 Institute of Ibnu Sina, Universiti Teknologi Malaysia, Johor 81310, Malaysia

**Keywords:** cell adhesion, attachment event, detachment event, adhesion strength

## Abstract

Cell adhesion is essential in cell communication and regulation, and is of fundamental importance in the development and maintenance of tissues. The mechanical interactions between a cell and its extracellular matrix (ECM) can influence and control cell behavior and function. The essential function of cell adhesion has created tremendous interests in developing methods for measuring and studying cell adhesion properties. The study of cell adhesion could be categorized into cell adhesion attachment and detachment events. The study of cell adhesion has been widely explored via both events for many important purposes in cellular biology, biomedical, and engineering fields. Cell adhesion attachment and detachment events could be further grouped into the cell population and single cell approach. Various techniques to measure cell adhesion have been applied to many fields of study in order to gain understanding of cell signaling pathways, biomaterial studies for implantable sensors, artificial bone and tooth replacement, the development of tissue-on-a-chip and organ-on-a-chip in tissue engineering, the effects of biochemical treatments and environmental stimuli to the cell adhesion, the potential of drug treatments, cancer metastasis study, and the determination of the adhesion properties of normal and cancerous cells. This review discussed the overview of the available methods to study cell adhesion through attachment and detachment events.

## 1. Introduction

Adhesion plays an integral role in cell communication and regulation, and is of fundamental importance in the development and maintenance of tissues. Cell adhesion is the ability of a single cell to stick to another cell or an extracellular matrix (ECM). It is important to understand how cells interact and coordinate their behavior in multicellular organisms. In vitro, most mammalian cells are anchorage-dependent and attach firmly to the substrate [1]. According to the “cell adhesion model”, the more a cell sticks the more it shows the greater number of chemical bonds it has on its surface [2,3].

Cell adhesion is involved in stimulating signals that regulate cell differentiation, cell cycle, cell migration, and cell survival [4]. The affinity of cells to substrate is a crucial consideration in biomaterial design and development. Cell adhesion is also essential in cell communication and regulation, and becomes of fundamental importance in the development and maintenance of tissues. Changes in cell adhesion can be the defining event in a wide range of diseases including arthritis [5,6], cancer [4,7,8], osteoporosis [9,10], and atherosclerosis [11,12]. Cell adhesiveness is generally reduced in human cancers. Reduced intercellular adhesiveness allows cancer cells to disobey the social order, resulting in destruction of histological structure, which is the morphological hallmark of malignant tumors [8]. Tumor cells are characterized by changes in adhesivity to ECM, which may be related to the invasive and metastatic potential. Alterations in cell-matrix and cell-cell interactions are cell type- and oncogene-specific. For example, while the transfection of rodent fibroblast cells with *Src* and *Ras* oncogenes reduces the adhesiveness to fibronectin (Fn) by impairing α5β1 integrins, the activation of oncogene *ErbB2* in breast cancer up-regulates α5β1 integrin and enhances adhesion [13,14]. The adhesion of highly invasive cancer cells altered the biomechanics of endothelial cells [15]. Mierke [15] reported that *MDA-MB-231* cells’ attachment may lower the endothelial cells’ stiffness by breaking down the cells’ barrier function through remodelling of the actin cytoskeleton.

Different requirements for cell adhesion are needed for various types of applications, and are dependent on the cell’s specific applications [16]. Various techniques to analyze cell adhesion have been applied to understand different fields of study including biomaterial studies [17], the effects of biochemical treatments and environmental stimuli to the cell culture [18], and determination of adhesion properties of normal and cancerous cells [19]. Biomaterials designed in biomedical engineering that have to interact with blood, like those in artificial heart valves or blood vessels, are required not to be adherent to cells or plasma proteins to avoid thrombosis and embolism. On the other hand, materials used in scaffolds for tissue generation are needed to act as substrate to promote the cells’ adhesion, subsequent proliferation, and biosynthesis [16]. Adhesion between cells allows blood clot formations that may lead to heart failure by restricting the blood supply to the heart muscles [16].

### 1.1. Focal Adhesion

**Figure 1:**
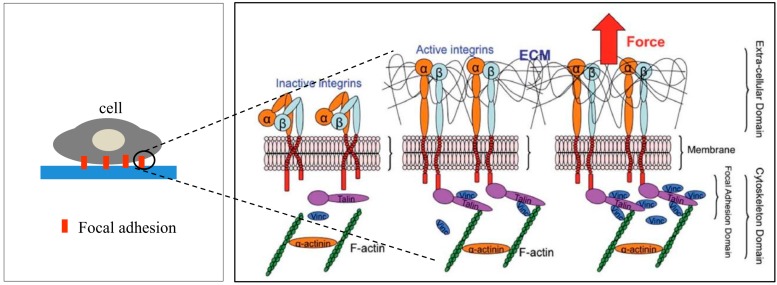
Schematic representation of activated integrin and formation of ECM-integrin-cytoskeleton linkages in the focal adhesion site upon application of an external tensile load. Reproduced “in part” from [24] with permission of The Royal Society of Chemistry.

Cells transmit extracellular or intracellular forces through localized sites at which they are adhered to other cells or an extracellular matrix. The adhesion sites are formed by transmembrane proteins called integrins to anchor the cell to a matrix or adhesion molecules to other cells [20]. Both the integrins and adhesion molecules are attached to the tensile members of the cytoskeleton, the actin filaments, through the focal adhesion (FA) complex (Figure 1), a highly organized cluster of molecules [21]. The cytoskeletal structure holds the nucleus and maintains the shape of the cell [22,23,24]. As a pathway for force transmission to the cytoskeleton, integrins play an important role in mechanotransduction through FA proteins connecting the integrin domains to the actin filaments to form the adhesion complex [24]. Upon binding, integrins cluster into FA complexes that transmit adhesive and traction forces [25,26]. The FA formation is important in cell signaling to direct cell migration [27], proliferation, and differentiation [28,29] for tissue organization, maintenance, and repair [28].

### 1.2. Phases of Cell Adhesion and Spreading

#### 1.2.1. Passive *in Vitro* Cell Adhesion

Passive *in vitro* cell adhesion is the cell adhesion process in a static medium culture, e.g., culture flasks, petri dishes. During static *in vitro* cell-matrix attachment and spreading, cells undergo morphologic alterations driven by passive deformation and active reorganization of the cytoskeleton. Integrin receptors and heterodimeric transmembrane proteins play a central role in cell adhesion and spreading. Specific integrin binding provides not only a mechanical linkage between the intracellular actin cytoskeleton and ECM, but also the bidirectional transmembrane signaling pathways [29,30,31,32,33]. Integrins recognize soluble ligands and insoluble ECM proteins and their interaction regulates cell responses such as cytoskeleton formation. The binding of integrins with their ECM proteins activates the Rho *GTPase* family (including *Rho*, *Rac*, and *Cdc42*), which is involved in cell spreading and migration [34,35], and *Rho* controls stress fiber formation and the assembly of focal adhesions [34].

The process of static *in vitro* cell adhesion is characterized by three stages (Table 1): attachment of the cell body to its substrate (initial stage), flattening and spreading of the cell body, and the organization of the actin skeleton with the formation of focal adhesion between the cell and its substrate [35]. Cell spreading appears to be accompanied by the organization of actin into microfilament bundles. The strength of adhesion becomes stronger with the length of time a cell is allowed to adhere to a substrate or another cell. The initial adhesive interaction between the cells and the substrate are driven by the specific integrin-mediated adhesion and starts with the binding of single receptor-ligand pairs [36]. This will initiate the subsequent receptor-ligand bonds and quickly enhance in number, thus increasing the total adhesion strength [37]. The adhesion properties of cells could be determined by studying various cell-substrate contact times [38].

Following initial attachment, cells continue flattening and spreading on the substrate, resulting in the decrement of cell height (the cell flattens) and increment of contact area (Phase I) [16]. Next, the cell spreads beyond the projected area of the unspread spherical cell (Phase II) [36]. The spreading process is the combination of continuing adhesion with the reorganization and distribution of the actin skeleton around the cell’s body edge [16]. Cells will reach their maximum spread area through expansion and adhesion strength will become stronger (Phase III).

**Table 1:** Evaluation of passive *in vitro* cell adhesion intervention and stages [36].

Cell Adhesion Phases	Phase I	Phase II	Phase III
Schematic diagram of cell adhesion	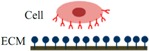	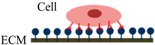	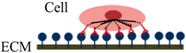
Schematic diagram of the transformation of cell shape	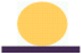 Initial attachment	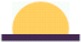 Flattening	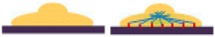 Fully spreading and structural organization
Cell adhesion intervension	Electrostatic interaction	Integrin bonding	Focal adhesion
Adhesion stages	Sedimentation	Cell attachment	Cell spreading and stable adhesion

#### 1.2.2. Dynamic *in Vivo* Cell Adhesion

The adhesion of cells to the extracellular matrix *in vivo* under blood flow is a dynamic process. Cells will undergo dynamic adhesion alterations during their morphogenesis, tissue remodeling, and other responses to environmental cues [39]. *In vivo* dynamic cell adhesion is mediated through molecular bonding between cell-surface receptors and their ligands or counter-receptors on the other cell surfaces in the extracellular matrix. Shear flow is a crucial factor to initiate cell adhesion as it mediates the activation of β-integrin via E-selectin signaling [40]. The adhesive bond is defined as the sum of non-covalent interactions, e.g., hydrogen bonds, electrostatic interactions, van der Waals forces, dipole-dipole interactions between two macro molecules [39]. Leukocytes or hematopoietic progenitors and tumor cells are the cells involved in dynamic vascular cell adhesion *in vivo*. Leukocytes and hematopoietic progenitors are the essential cells in the human immune response system that will migrate from one site to another to provide the effector function. Tumor cell interactions with endothelium and the subendothelial matrix constitutes a crucial factor in determining the metastatic potential of the cells and organ preferences of cancer metastasis [41,42].

**Figure 2:**
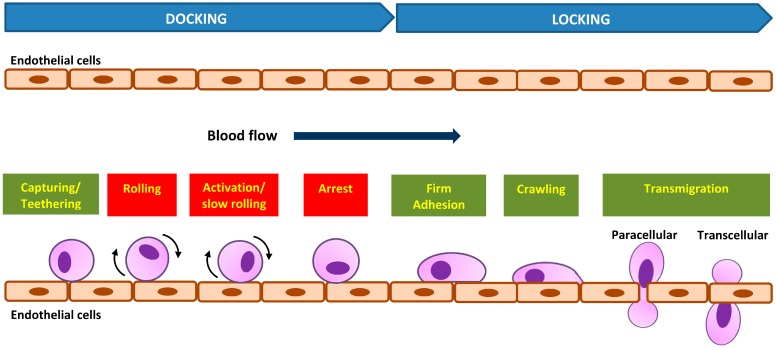
Dynamic *in vivo* cell adhesion cascade with “docking” and “locking” phases. The basic cascade steps are labeled in red boxes and steps recovered later are labeled in green boxes.

The cell adhesion cascade and signaling events *in vivo* involve three basic steps: selectin-mediated rolling, chemokine-triggered activation, and integrin-dependent arrest [43]. Initial recognition of dynamic cell adhesion *in vivo* involves the “docking” phase, which occurs between the rolling of cells to endothelial cells and to cell arrest (Figure 2), mediated by a weak and transient adhesion mechanism involving carbohydrate-carbohydrate and/or carbohydrate-protein interactions. The molecules involved at this stage are cell-surface conjugates, selectins, chemokines, or immunoglobulins (Igs) [42]. The cell adhesion cascade begins as a cell tethers to roll on the vessel’s wall. Molecular bonding between the adhesion molecules must form rapidly for cells to tether, and the bonding must break rapidly for cells to roll [39]. The rolling cells transduce signals from adhesion receptors and chemokine receptors that cause cells to roll slower and then to arrest, a prerequisite for emigration through the vasculature into underlying tissue [39].

Subsequently, cells will established stable bonds with endothelial cells during the activation-dependent “locking” phase, mediated largely by integrins and modulated by a host of bioactive mediators resulting from the activated cells [42]. Integrin-mediated adhesion is characterized by at least two events: arrest from rolling, which is mediated by increased cell avidity to endothelium, and a post-binding phase of adhesion stabilization [43]. In the “locking” phase, cell adhesion strengthening and spreading happens similarly to the static *in vitro* adhesion followed by intravascular crawling and transmigration (paracellular or transcellular) (Figure 2) [43]. This then permits the adhered cells to emigrate out of the vasculature. The intraluminal crawling facilitates the cell adhesion to emigration [44]. The post-adhesion events strengthen cell attachment to the endothelium, and into the molecules that are involved in cells’ transendothelial migration [45,46].

## 2. Types of Adhesion Studies

**Figure 3:**
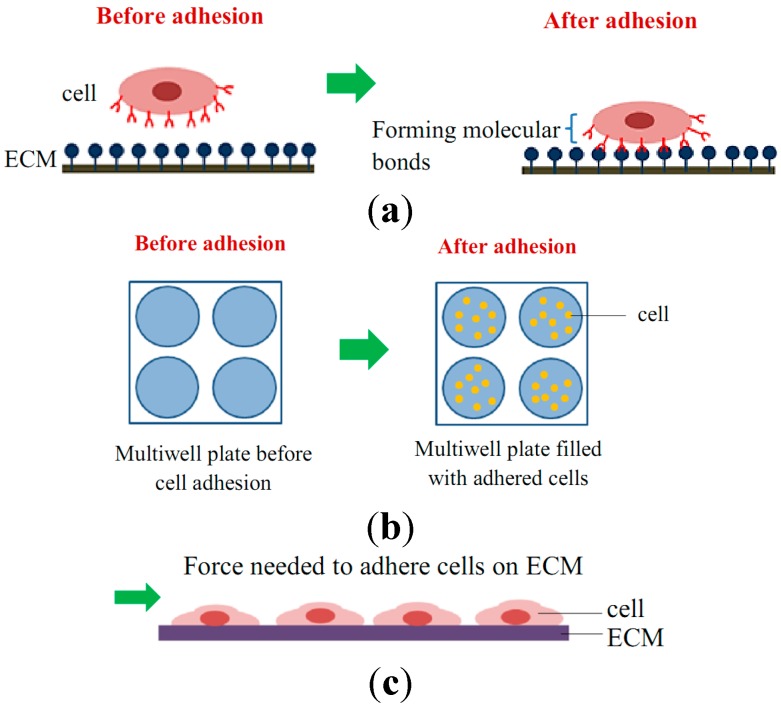
Schematic diagram of cell adhesion attachment events for (**a**) single cell studies via the formation of molecular bonds; (**b**) cell population studies via static adhesion (e.g., wash assay technique); and (**c**) cell population studies via dynamic adhesion (e.g., microfluidic technique).

**Figure 4:**
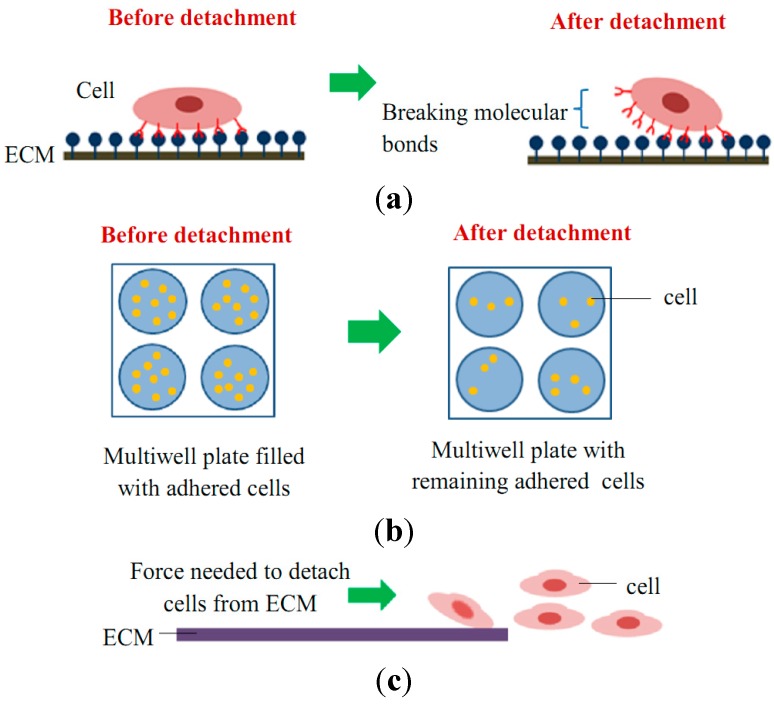
Schematic diagram of cell adhesion detachment events for (**a**) single cell studies via the breakage of molecular bonds (e.g., SCFS, micropipette aspiration, and optical tweezer techniques); (**b**) cell population studies via static adhesion (e.g., centrifugation technique); and (**c**) cell population studies via dynamic adhesion (e.g., spinning disk, flow chamber, and microfluidic techniques).

The mechanical interactions between a cell and its ECM can influence and control cell behavior and function. The essential function of cell mechanobiology and its progressively important role in physiology and disease have created tremendous interests in developing methods for measuring the mechanical properties of cells. In general, cell adhesion studies can be categorized into cell attachment and detachment events. Numerous techniques have been developed to analyze cell adhesion events through the study of single cells as well as the populations of cells. Cell adhesion attachment events are focusing on the cell attachment mechanism to the substrate, while the detachment events involve the application of load to detach the adhered cells on the substrate (Figure 3 and Figure 4).

### 2.1. Cell Adhesion Attachment Events

**Figure 5:**
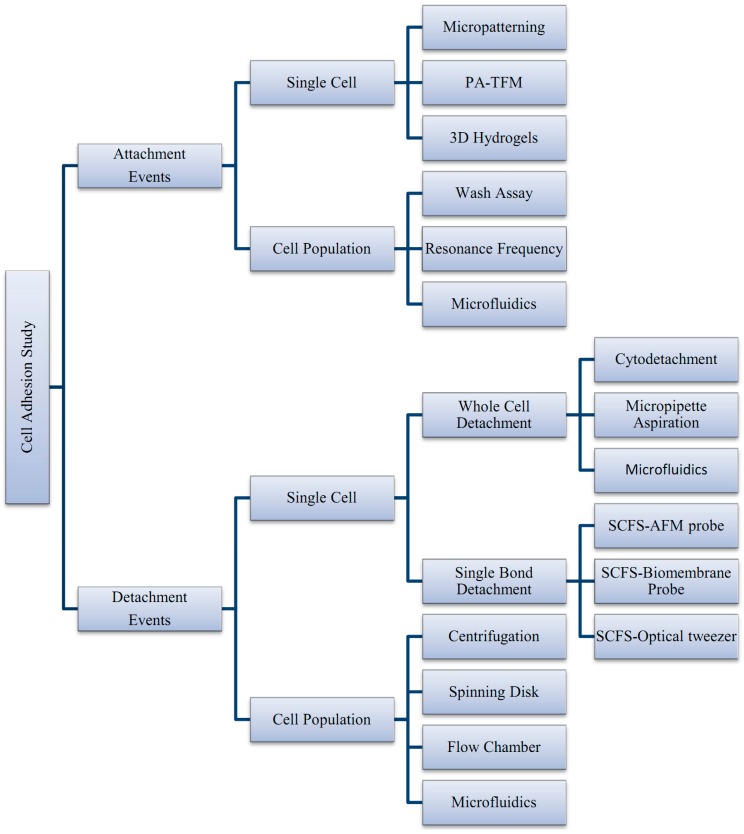
Summary of techniques involved in cell adhesion studies, categorized by the adhesion attachment events and detachment events.

Cell attachment studies cover the analysis from the formation of a molecular bond between the cell’s surface receptors and the complementary ligands (on the ECM’s surface) to the observation of a population of cells’ responses through the cells’ behavior and changes of morphology during the attachment events. In cell migration, the cell adhesion plays a pivotal role in the driving force production [27]. The adhesion events could be grouped into single cell and cell population analysis (Figure 5). For the single cell study, the experiments were performed to analyze the interaction forces between the individual cell and its substrate (Figure 3a), observing the individual cell’s morphology changes, studying the cell’s migration, and measuring the cell’s traction forces using polyacrylamide (PA) gel-based traction force microscopy (PA-TFM), micropattering technique, and three-dimensional traction force quantification (3D-TFM). Population studies involve the analysis of attachment events for a group of cells. It is important in analyzing the adhesion behavior of cells toward treatments or different physiological conditions (e.g., wash assay and microfluidic techniques), for the understanding the cell adhesion kinetics (e.g., resonance frequency technique), in determining the biocompatibility of biomaterials for tissue engineering, cancer metastasis studies, and also the potential of drug treatments (e.g., microfluidic techniques).

#### 2.1.1. Techniques to Study Cell Attachment Events

##### Attachment Events: Single Cell Approach

**Polyacrylamide-Traction Force Microscopy (PA-TFM).** Numerous techniques have been developed to understand cell adhesion by characterizing single cells during their attachment events. PA-TFM is one of the widely used techniques to study single cells’ traction force, the force exerted by cells through contact to the substrate surface. Cells will be cultured on the polyacrylamide gel functionalized with the cells’ adhesive ligands and fluorescent beads embedded near the gel surface [47]. Upon the adhesion attachment events, cells will generate traction forces that move the beads and the movement will be quantified by tracking the displacement of the fluorescent beads [47,48,49]. Reinhart-King *et al.* [50] reported cell spreading increased with the increasing concentration of arginine-glycine-aspartic acid (RGD)-peptide by measuring the magnitude, direction, and spatial location of mechanical forces exerted by endothelial cells. Sabass *et al.* [51] improved the reliability and spatial resolution of traction force microscopy to 1 um by combining the advances of experimental computational methods. An epithelial wound-healing assay was developed by Ng *et al.* [52] to study the migration of individual *MCF10A* on PA substrates with a range of substrate compliances. Their findings showed that the wound could initiate a wave of motion that directs cells’ coordination towards the wound edge and substrate stiffness influenced the collective cell migration speed, persistence, and directionality as well as the coordination of cell movements [52]. Traction forces of human metastatic breast, prostate, and lung cancer cell lines were found to be higher than non-metastatic counterparts, suggesting the cellular contractile force involve in metastasis and the physiological environment might regulate cellular force generation [48]. Wen *et al.* [53] reported stem cell differentiation was regulated by the stiffness of planar matrices independently of protein tethering and porosity. Beside single cell analysis, there have also been some adhesion studies done on the population of cells. Endothelial cells were reported to exert greater traction forces compared to single cells once in contact with the adjacent cell, thus suggesting an increase in cellular contractility with contact [54].

**Micropatterning.** Micropatterning (also known as microfabricated elastomeric post array (mPADs) or micropillar) is a method that provides a micrometer scale: a soft, three-dimensional complex and dynamic microenvironment for both single cell studies and also for the multi-cellular arrangements in populations of cells. It relies on basic elastic beam theory, which makes force quantification easier and more reliable, as there is only one traction force field for each micropost/micropillar displacement map [55]. Cell micropatterning comprises the fabrication and use of a culture substrate with microscopic features that impose a defined cell adhesion pattern. It is an efficient method to investigate the sensitivity and response of a cell to specific microenvironmental cues [56]. At the basic level, micropatterning approaches involve controlling cellular attachment, shape, and spreading as a function of the engineered spatial properties of the cultured surface [57]. Micropattering could be used to study cell adhesion for both the single cell level and also for the population of cells. Tan *et al.* [26] found that cell morphology regulates the magnitude of traction force generated by cells. These findings demonstrate a coordination of biochemical and mechanical signals to regulate cell adhesion and mechanics, which introduce the use of arrays of mechanically isolated sensors to manipulate and measure the mechanical interactions of cells [26]. Mandal *et al.* [58] introduced the micropatterned surfaces combined with the thermo-responsive poly(*N*-isopropyla-crylamide) (PNIPAM) as an actuator which induces cell detachment when the temperature is reduced below 32 °C. It has been reported that the micropatterning technique is able to independently tune the biochemical, mechanical, and spatial/topography properties of biomaterials that could provide the opportunity to control cell fate for tissue engineering and regenerative medicine applications [59]. Laminar atheroprotective fluid shear stress has been found to induce increments in traction force generation and endothelial cell alignment, which are associated with inflammation and atherosclerosis progression [60].

**Three-Dimensional Traction Force Quantification (3D-TFM)**. The ability to grow cells within ECM gels (3D culture) is a major advantage to understand *in vivo* cellular cell behaviors, ranging from differentiated function to maintenance of stem cell functions [61,62]. The 3D-TFM technique uses 3D matrixes such as agarose, collagen, hyaluronic acid, fibrin, or matrigel for the cell culture. Individual cells are grown inside the gel matrix embedded with fluorescent beads surrounding the cell. Bead dispersion in the 3D gel will be observed to estimate the cellular contractility of the cell during migration [63,64,65,66,67]. In contrast to 2D migration, cell migration through a dense 3D network of extracellular matrix proteins is possible only when the cell generates sufficient tractions to overcome the steric hindrance of the surroundings [68]. Koch *et al.* [69] used the method to develop the 3D traction map of several tumor cell lines (*MDA-MB-231* breast carcinoma, *A-125* lung carcinoma) and found that the directionality is important for cancer cell invasion rather than the magnitude of traction, and the invasive cells elongated with spindle-like morphology as opposed to the more spherical shape of non-invasive cells [69]. The disruptive effect of the nocodazole drug on the neuronal processes has been analyzed using matrigel-embeded microbeads and neuron cells [65]. Kutys and Yamada [70] managed to explored pathways controlling the migration involving the GEF/GAP interaction of *βPix* with *srGAP1* that is critical for maintaining suppressive crosstalk between *Cdc42* and *RhoA* during 3D collagen migration. Fraley *et al.* [66] reported that instead of forming aggregates in the 3D matrix, the focal adhesion proteins diffused and distributed throughout the cytoplasm and were responsible in modulating cell motility.

##### Attachment Events: Population Approach

**Wash Assay.** In the population cell adhesion studies, the process of cell attachment can be divided into two types; static culture and dynamic culture, depending on the cell adherence mechanism during the cell culturing. Static culture is the stagnant condition of the cell culture medium during the incubation for cell adhesion, which is applicable for the culturing of cells inside microwell plates (Figure 3b), petri dishes, culture flasks, and cell cultures on the ECM-coated cantilever inside the chamber. The static culture was used in the wash assay and resonance frequency techniques. In the wash assay, cells were cultured in 96 multiwell plates for the cell attachment events, followed by cell washing before adhesion analysis (e.g., cell count, protein/DNA count, or antibody binding) was carried out [71,72,73,74]. Wash assays provide basic qualitative adhesion data by determining the fraction of cells which remain adhered after one or more washings [75]. Cells that remain adhered to the substrate after washing will be analyzed for further quantitative analysis such as cell count, quantification of DNA content, protein count, or antibody binding. Treatment of *D. mucronata* crude extract to the cancerous *wehi-164* cells significantly modulated their attachment and spreading behavior to the fibronectin-coated multiwell plates [74]. Chen *et al.* [73] extracted the adhered HeLa cells from the collagen-coated multiwell for further enrichment process and analysis. Park *et al.* [71] developed adhesion-based assay for high-throughput screening for the discovery of small-molecule regulators of the integrin CD11b/CD18 to further understand the mechanism of integrin activation and binding.

**Resonance Frequency.** The integration of advanced microelectronics technology with signaling processing and biological sensing interfaces has grown widely to develop biosensor devices. Piezoelectric sensor is the acoustic sensor which is able to detect label-free and selective biological events in real time. Quartz crystal microbalance (QCM) is one of the widely used piezoelectric acoustic wave resonator [76] biosensors for the study of cell adhesion and cell spreading. It is comprised of a thickness shear mode resonator made from a thin (AT-cut) quartz crystal sandwiched between two metal electrodes [77]. The sensor is coated with ECM before cells are mounted on it and placed in the chamber for cell adhesion to occur. Whole cells will act as sensing elements in the cell-based biosensors, as cells continuously react to the environment. The piezoelectric resonators will perform shear oscillations parallel to the sensor faces [78] upon cell adhesion activity, which will propagate through the sensor in a direction perpendicular to its surface. During attachment and cell spreading on the sensor surface, changes in resonant frequency could be detected upon the interactions between the cell membrane and the substrate, and upon the changes in the fractional surface coverage by the cells [77,78,79,80,81,82,83,84,85]. The resonance frequency of the sensor alters when a foreign mass attaches to the sensor’s active surface and the frequency shifts will represent the mass of the absorbed material [77]. The adhesion process and the molecular interactions will produce signals representing cell adhesion kinetics determined by the sensor [36,83,86,87]. This technique has been found able to monitor cell attachment and spreading of animal cells on a particular surface quantitatively and in real time.

Zhu *et al.* [87] found the mechanisms governing elasticity and adhesion are coupled and affected differently during aging. Heitmann and Wegener [78] reported that the resonance frequency technique not only can be used to monitor cell adhesion but can also present as an actuator to perturb cell-substrate contact sites without causing damage to the cell when using the driving voltage for normal monitoring amplitude. The propagation properties of the acoustic wave in materials vary according to the different properties of the materials, and their energy is dissipated in the presence of fluids or viscoelastic materials [76,88]. The variation of the sensor resonance frequency (∆*f*) and acoustic wave energy dissipation can be used to gain direct measurements of the physical properties of the layers in contact with the chip [76,88,89]. The obvious difference in total frequency shift is caused by the different numbers of attached cells, since the geometries and the fundamental resonance frequency are similar. Besenbacher *et al.* [84,85] analyzed cell adhesion and spreading of *MC3T3-E1* and *NIH3T3* cells on different precoated biocompatible surfaces. Braunhut *et al.* [90,91,92,93] have developed and optimized the performance of the QCM biosensor and carried out a study on the effects of various types of anti-tumor drugs (e.g., nocodazole, taxol, taxane) on the adhesion of different types of cancer cells. In the advancement of resonance frequency technology, the sensor has been shown capable of acting as both sensor and actuator. It can be used as a sensor for cell adhesion and as an actuator to induce oscillations on the growth surface [78]. Recently, the usage of resonance frequency technique in cell adhesion has emerged to provide a platform for small sample sizes of cells in a dynamic fluid condition. Resonance frequency technique is used as an actuator to provide the acoustic wave needed for the dynamic fluid condition in the device and as sensor to measure cell adhesion. Warrick *et al.* [94] have developed a high-content adhesion assay to overcome the limitation of the available methods to perform analysis on small animal biopsies and rare cell isolations. Hartmann *et al.* [95] produced a new tool for the dynamic analysis of cell adhesion that provides small cells’ sample volume, a short measuring time, and flexibility for different types of substrates that are suitable for studying the implant material compatibility.

**Microfluidics**. In contrast to the static culture, the dynamic culture applies fluid movement during the cell culturing and adhesion process. Low fluid shear flow is needed to help the cell attachment process as it mimics the blood flow in the human body. Cells are continuously exposed to hemodynamic forces generated by blood flow in most biological systems. The balance between the adhesive forces generated by the interactions of membrane-bound receptors and their ligands with the dispersive hydrodynamic forces determines cell adhesion [96]. Cell adhesion attachment events dynamic culture can be observed using microfluidic technique. The advantages of microfluidic systems (fluid manipulation and control, low fluid intake and miniaturization) encouraged their use in dynamic culturing for cell adhesion studies. This technique was used to study the ability of cells to adhere and to observe cell spreading, tracking, and migration inside the channel under the influence of fluid flow. Rupprecht *et al.* [97] reported that cell shape, movements, and the rate of cell division were found to be similar in petri dishes and microfluidic channels. Alapan *et al.* [98] applied the microfluidic technique to analyze the adhesion properties and deformability of human red blood cells in flow using blood samples from 12 different subjects. From the study of cell adhesion properties and dynamics, the technology has grown and been upgraded into the development of tissue-on-a-chip and, later, organ-on-a-chip for biomedical and pathological studies. Cell monolayers were grown in the microfluidic channel and made to mimic the human vascular system to be used for important bioengineering and biomedical analysis. A multi-step microfluidic device has been developed by Chaw *et al.* (2007) to analyze the deformation and biological and migratory capability of various tumor cell lines (*HepG2*, *HeLa*, and *MDA-MB 435S*) to the lining of *HMEC* cells inside the channels [99]. Nalayanda *et al.* [100] have built a series of bio-mimetic devices for the model of alveolar-capillary membranes while Song *et al.* [101] have developed a cancer cell metastasis model using microfluidic technology. Fu *et al.* [102] studied tumor cell adhesion to the endothelium cell layer under shear flow by combining micro-particle imaging velocimetry (μPIV) technique with flow chamber assay to understand the interactions between leukocytes and tumor cells near the endothelium wall region. A microfluidic model for organ-specific extravasation of circulating tumor cells (CTCs) has been build by Riahi *et al.* [103] to demonstrate the extravasation of *MDA-MB-231* cancer cells by analyzing the cells’ adhesion capability to the endothelial monolayer inside the channel.

### 2.2. Cell Adhesion Detachment Events

Cell adhesion detachment studies involve load application to the adhered cells on the ECM to free the cells from their cell-matrix bonding (Figure 4). The applied force that produces cell detachment is quantified as the cell’s adhesion strength. Many types of assays have been developed to measure cells’ adhesion strengths and can be divided into single cell and population cell studies (Figure 4b,c). Measuring cells’ adhesion strengths has become an emerging interest in various areas of study, including biomaterial compatibility in the human body, characterizing different stages of cancer cells, drug treatments for diseases, and the discovery of biomarkers for early disease diagnosis. Cells will be cultured and allowed to adhere on the ECM-coated matrix followed by cell detachment processes and adhesion strength measurement. For the single cell approach, the detachment process is focused on an individual cell and the measured value represents the adhesion strength of a single cell. Single cell detachment techniques were carried out either to fully detach a single cell from its substrate (whole cell detachment) for obtaining a single cell’s adhesion strength or to focus on the load needed for breaking the molecular bonds to further understand cell adhesion kinetics. Techniques used for whole cell detachment are the cytodetachment and micropipette aspiration techniques, while to perform molecular bond breakage, the single cell force spectroscopy (SCFS) technique was used (Figure 4). Cell detachment events for the cell population approach were carried out by applying load at the population on adhered cells. Following the detachment process, the fraction of cells remaining on the substrate is quantified after varying loads of global force or stress have been applied. The force or stress value at which 50% of the cells detach is determined as the population adhesion strength. The adhesion measurement techniques for the cell population approach can be divided into four categories depending on the loading method applied to detach cells: centrifugation assay, spinning disk, flow chamber, and microfluidics (Figure 5).

#### 2.2.1. Techniques to Study Cell Detachment Events

##### Single Cell Approach

**Cytodetachment**. Cytodetachment technique uses an atomic force microscopy (AFM) probe to physically detach individual cells in an open medium environment such as petri dish [75]. Cells have to be attached to a functionalized matrix before the single cell-probe alignment and probe translation application are carried out to detach the cell. Force is quantified by measuring the elastic deflection of the probe used to detach the cell and then divided by the cell area to calculate the average shear stress for each cell [75]. Yamamoto *et al.* [104] studied the force needed to detach murine fibroblast *L929* on four different materials by using cytodetachment technique. Using an image analysis system and fiber optic sensor, the apparent cell adhesive area was measured and the adhesive strength and detachment surface energy were calculated by dividing them by the cell adhesive area [104]. Their findings showed that cells on collagen-coated polystyrene produce the highest adhesion strength and cell detachment surface energy, and the adhesive properties of the cells between both polystyrene and glass are almost the same [104]. Human cervical carcinoma cells (*NHIK 3025*) were found to attached stonger and faster to the hydrophilic substrate using technique 1 at 37 °C when compared to 23 °C. There are multiple adhesion measurement studies combining the cytodetachment method with the laser tweezer work station [105] and optical tweezer technique to further understand the temporal effects of cell adhesion by analyzing the molecular binding between the cell and the substrate [16,105]. The study of bovine articular chrondocyte cell adhesion with different ECM-coated substrate was carried out by using a combined cantilever glass beam with a carbon filament as a cytodetacher. Huang *et al.* [16] used rabbit articular chrondocyte cells to study the mechanical adhesiveness of the cells using the cytodetachment method. They examined membrane cell tether formation using optical tweezers and cytoskeleton change through fluorescent staining. Findings showed that chrondocytes exhibited increasing mechanical adhesiveness and tether formation force with the increment of seeding time. Yang *et al.* [106] reported that the adhesion force of human fetal osteoblast (*hFOB*) cells was influenced by the cell’s shape grown on the Ca-P grooved micropattern surface.

**Micropipette Aspiration Technique**. Micropipette aspiration is a widely used technique for measuring the mechanical properties of single cells. For single cell adhesion measurement, this technique detaches an immobilized cell by applying suction force to a portion of the cell surface employed by micropipette suction [107] under observation via a microscope. The force will release the cell from the substrate by increasing the aspiration pressure or by translating the pipette away from the substrate [75]. Adhesion strength is defined as the minimum force needed to detach a single cell from its substrate. Micropipette aspiration technique is able to measure various mechanical properties of cells, such as the membrane stiffness of chondrocytes and endothelial cells [108], the cortical tension of neutrophils [108], and the adhesion strength of human umbilical vein endothelial cells on different substrates [109]. Gao *et al.* [109] used cells in the phase they called the “adhered cells in round shape” phase to eliminate the influences of floating cells, weakly adhered cells, and spread cells and obtained high reproducibility and sensitivity of the measurement on different substrates. They reported that the sensitivity and accuracy of their technique could reach 8 × 10^−^^12^ N [109]. Micropipette aspiration technique was found able to identify comparable differences between the adhesion strength of normal and cancerous cells. Palmer *et al.* [18] have developed a single cell measuring apparatus using micropipette aspiration (SCAMA) that is able to measure and differentiate the adhesion strength of normal and cancerous prostate and breast cells. They found the measurement made on analogous human prostate cancer and normal cells showed a comparable three-fold difference in adhesiveness [18].

**Single Cell Force Spectroscopy (SCFS)**. Force spectroscopy measurement methods were developed to measure the strength of cell adhesion down to single cell level. Commonly, the methods will use a microscope to observe the cell while force is applied to detach the cell using a nano/micromanipulator or micropipette. The imaging mode is used to study the structures and mechanics of isolated biomolecules [110,111,112], components of the cell nucleus [113,114], and subcellular cytoskeletal structures [110,115], while force mode is used to determine the mechanical properties of various cell types. The methods differ in the type of manipulation applied to the cell and the type of force measurements made. Examples of single cell force spectroscopy techniques used to measure cell detachment are the AFM probe techniques, biomembrane probe (BFP), and optical tweezer methods. Among these methods, AFM-based techniques are widely used to study various types of cell adhesion, effects of surface treatments and environmental conditions, and biomaterials compatibility.

**AFM Probe Force Measurement**. AFM probe force measurement is widely used to measure the stiffness [116,117,118,119] and adhesion strength of individual cells against mechanical force [16,104,105,120] due to its versatility and precision. By immobilizing individual cells to an AFM cantilever, the living cell will be converted into a probe for the measurement of adhesion strength between cell-cell or cell-matrix adhesions [38]. This probe is attached to a cell or ECM-coated substrate (cell adhesion occurs) and the cantilever is withdrawn at a constant speed to detach the cell from its binding place. Cantilever deflection is recorded as force-distance curve and the highest force recorded represents the cell’s adhesion strength [38]. When the probe encounters the single cell surface, various forces between the cantilever probe and cell lead to a deflection of the cantilever according to Hooke’s law [120]. Adhesion strength of a single cell could be monitored as a function of adhesion time and environmental conditions by using AFM probe force measurement [75]. Various aspects of adhesion could be studied using AFM probe SCFS without restriction on the type of cell adhesion molecules and cell types used. Puench *et al.* [121] reported that the technique is able to quantitatively determine the adhesion of primary grastulating cells and provide insight into the role of *Wnt11* signaling in modulating cell adhesion at single cell level. Weder *et al.* [122] studied the adhesion of human osteosarcoma cell lines (*Saos-2*) during different phases of the cell cycle and found that the cells are loosely attached to the substrate during the cells’ round up (*M* phase) compared to during the interphase. Hoffmann *et al.* [123] determined the influence of the activating *NK* cell receptor 2B4 on the early adhesion processes of *NK* cells using AFM-probe SCFS. In addition, AFM is flexible and can be integrated with the standard modern inverted and transmission optical microscope. Lee *et al.* [105] developed a cell-detachment apparatus to measure the adhesion force of single cells integrated with a laser tweezer work station for cell manipulation observation. They studied the effect of experimental medium on the cell adhesion force of *NIH/3T3* fibroblast cells and found the cell adhesion strength increased with culturing time, and growth factor was found to enhance the adhesion strength between the cell and substrate. The study was continued with the combination of the optical trapping technique to further study the single cell adhesion properties of *MCDK* cells in different phases of adhesion [119]. Their findings showed that focal adhesion kinase (FAK) plays a role in enhancing the binding and spreading of *MDCK* cells through all the different phases of cell adhesion. Beaussart *et al.* [124] studied the adhesion forces of medically important microbes using AFM-SCFS and showed that procedures are applicable to pathogens such as *Staphylococcus epidermidis* and *Candida albicans*.

**Biomembrane Force Probe (BFP)**. BFP is a sensitive technique that allows the quantification of single molecular bonds. It is a versatile tool that can be used in a wide range of forces (0.1 pN to 1 nN) and loading rates (1–10^6^ pN/s) [125]. This technique uses a force transducer made of a biotinylated erythrocyte (such as biotinylated red blood cell (bRBC)) maintained by a glass micropipette. A streptavidin-coated glass microbead was attached to the bRBC (with known stiffness) and tuned by the controlled aspiration pressure applied by the holding micropipette. The assembly formed by the bRBC and the microbead constitutes a powerful nanodynamometer and is the force transducer (probe) used in the BFP [125]. The probe is brought into contact with the targeted cell and adhesion (bond formation) will occur between the probe and cell, followed by the detachment process where targeted cells are pulled away from the probe using a piezoelectric manipulator. Evans *et al.* [126,127] developed a transducer capable of measuring force ranging from 10^−2^ to 10^3^ picoNewton (pN) for probing molecular adhesions and structures of a living cell interface [126] to improve their previous probing method [127]. The BFP technique was used to quantify the human neutrophil (PMN) membrane unbinding forces and the kinetics rate for the membrane unbinding was found to increase as an exponential function of the pulling force [128]. Gourier *et al.* [125] proved the capability of the technique to quantify the local changes of gamate (oocyte and spermatozoan) membrane adhesion and probe the mechanical behavior of the oocyte membrane at a micrometer scale.

**Optical Tweezers (OT)**. OT uses a highly focused laser beam to trap and manipulate microscopic, neutral objects such as small dielectric spherical particles that experience two kinds of forces: namely, the scattering force and the gradient force [129,130]. The technique is able to measure forces ranging from sub-pN up to several hundreds of pN with good precision (<1 pN) and is applicable for the study of interfacial interactions and non-covalent bonds, e.g., receptor-ligand bonds [129]. Single cell adhesion studies have been explored using OT [131,132,133,134,135,136] involving various cell types and purposes. Askenasy and Farkas [137] used OT for studying the cellular adhesion of hematopoietic stem cells (HSC) to the bone marrow stroma, and a forward scatter analysis (FORSA) has been integrated with the OT to investigate the binding force associated with cell-cell interactions and molecular interactions [132]. Thoumine *et al.* [131] were able to produce information on the receptor-ligand adhesion kinetics of fibroblast cells to fibronectin by coupling the experimental results with a probabilistic model of receptor-ligand kinetics. They gained information on the number, strength, and reaction rates of the bonds. Optical tweezers have also been applied to study the adhesion of *Saccharomyces cerevisiae* cells [134,135,138] and to map the adhesion force during the formation and maturation of cell adhesion sites of mouse embryonic fibroblasts [136].

##### Detachment Events: Population Approach

**Centrifugation Assay.** Centrifugation assay is one of the frequently used techniques to measure cells adhesion strength due to their simplicity and the wide availability of equipment in most laboratories. Cells will be seeded in a multiwell plate and undergo treatments by culturing (cell culturing is similar to wash assay) before being spun for the cell detachment process. During the spinning, cells will experience a body force acting in the direction normal to the bottom of the plate that pulls them away from the surface [75]. To assess adhesion strength, the number of cells before and after application of load in the centrifuge is quantified. The fraction of cells that remains adhered after centrifugation can be determined by measuring the amount of radiation emitted from radio-labeled cells [139,140], quantifying the amount of cells or cellular genetic material [139], or by using automated fluorescence analysis [140,141]. In many cases, the assay is used to assess the relative effect of treatments such as ECM protein type and concentration or the inhibition of a specific cellular function.

This method was used by Channavajjala *et al.* [141] to understand the importance of cell attachment to HIV-1 Tat protein and their finding shows that Tat protein mediates a significant but weak attachment of *HT 1080* cells compared to the cells binding to ECM proteins. García *et al.* [142,143,144,145] used centrifugation assay to study cell adhesion and integrin binding and the effects of surface functionality of self-assembled. Harbers *et al.* [146,147] demonstrate the importance of flanking amino acids in the developing ligands with tuneable activity and the relative adhesion strength of each ligand by high-throughput assays for rapidly testing receptor-ligand engagement. High-throughput capabilities of the centrifugation method have been applied by Reyes and Garcia [144], who analyzed the adhesion capabilities of different cells (*HT-1080*, *NIH3T3* and *MC3T3-E1*) on different concentrations of collagen or fibronectin. Koo *et al.* [140] used the method to study the effect of different ligand density and clustering to show that biophysical cues such as ligand spatial arrangement and ECM rigidity are central to the governance of cell responses to the external environment.

**Spinning Disk**. The spinning disk technique utilizes shear stress generated from a rotating disk device. Cells are first seeded on circular glass coverslipsor on the surface of a disk (typical diameter 10–50 mm). These disks are later fixed onto a rotating device, that is placed inside a chamber filled with buffer solution [75,148]. The rotating device has a rotating range from 500 to 3000 rpm. The adherent fractions of cells are generally quantified using microscopy and by counting the number of cells before and after spinning using either a manual procedure [149] or automated image processing software [150]. The spinning disk technique has been used to investigate various types of cell-substrate interactions for wide range of applications such as quantifying the adhesion strength of an osteoblast-like cell on bioactive glass [150,151], human bone marrow cells on hydroxyapatite [152,153], and *MC3T3-E1* cells on RGD peptides on self-assembled monolayers [154]. Lee *et al.* [154] used this method to quantify the nonspecific and specific contributions of bone cells on immobilized RGD peptides and quantitatively demonstrated both the possibilities and limitations of enhancing the osteogenic response of RGD-immobilized biomaterials by a change in peptide. The method has been used to study the role of focal adhesion kinase, which is essential for the focal adhesion function and the cell’s adhesion strength [154,155]. Boettiger *et al.* observed the effect of transformed oncogene *v-src* on the adhesion strength of chick embryo fibroblasts [156] and human osteosarcoma cells [157] to understand its effect on integrin function. Lynch *et al.* [158] investigated the nature of signaling mechanisms that regulate integrin function in a steady-state adhesion and during cell motion using cells exposed to the insulin-like growth factor I (*IGF-I*). The effect of surface charges on different substrates has also been studied using *HT-1080* cells following fibronectin coating [159]. Reutelingsperger *et al.* [148] have investigated the effects of differential shear stresses on cell-cell and cell-matrix interactions in a monolayer of endothelial cells. García *et al.* [160] were able to measure the short- and long-term adhesion strength of *IMR-90* human fibroblasts adhered to fibronectin-coated glass using the spinning disk technique.

**Flow Chamber**. There are two types of flow chambers used for adhesion strength measurement: the radial and parallel flow chambers. The radial flow chamber methods involve flowing fluid in a chamber over adhered cells on a stationary substrate where a wide range of radially dependent shear stresses are applied [161] to detach a population of cells in a single experiment. The fluid flow is directed outwards from the center of a circular chamber, impinges on the surface of interest, and flows radially outward over a substrate seeded with cells. The inlet flow is directed outwards from the center of the chamber and the shear stress of the fluid decreases with increasing radial distance in a nonlinear fashion [75]. The radial flow chamber is also known as a stagnation point flow chamber or confined impinging jet [162]. The linear fluid velocity and shear stress will decreased radially across the disk as the flow duct cross-sectional area increases with the radius, and this method provides a continuous range of shear force in a single experiment [162].

The radial flow chamber technique has been used by Cozens-Roberts *et al.* [163] to investigate the effects of ligand and receptor densities and the influence of the pH and ionic strength of the medium on the cell-surface interactions. DiMilla *et al.* [164] studied the human smooth muscle cells (*HSMC*s) initial attachment strength and migration speed on a range of fibronectin and collagen type IV concentration. Their finding’s suggested that cell-substrate initial attachment strength is a central variable governing cell migration speed and the cell’s maximal migration occurs at an intermediate level of cell-substrate adhesiveness [164]. A number of studies have been carried out to study the adhesion strength of murine *3T3* fibroblasts on the fibronectin-coated of self-assembled monolayers on glass surfaces [161,165,166,167]. The adhesion of *MC3T3-E1* cells to multilayer polyallylamine hydrochloride (PAH) heparin films was analyzed in order to evaluate biocompatibility of various film chemistries [168]. Rezania *et al.* [169,170,171,172,173] carried out studies on the adhesion of osteoblasts and osteoblast-like cells to RGD peptides [169,170,171] and the adhesion of endothelial cells to interpenetrating polymer networks [173] for the application of orthopaedic implants. A study by Brown *et al.* [174] showed that low trypsin concentrations could improve cell adhesion and promote stronger endothelial adhesion while high trypsin concentrations significantly reduced the number of functional integrins available on the membrane. Beside human cells, the radial flow method has also been used to understand the adhesion kinetics of bacteria *Escherichia coli* K12-D21 in the in the mid-exponential and stationary growth phases under flow conditions. Chinese hamster ovary cells (*CHO*) were used to explore the adhesion behavior towards different substrates with different treatments [175].

The parallel plate flow chamber consists of a bottom plate and an upper plate separated by a distance of the channel’s height to form a rectangular flow channel. Cells are grown on a coverslip and positioned in the flow chamber, constructed by sandwiching a thin rubber gasket between two plates and mounted on a microscope to allow direct observation of the cells during experiments [75]. The flow is often driven using hydrostatic pressure from a raised reservoir or with an automated pump to independently drive the flow [176,177,178]. The fluid’s shear stress can be adjusted by varying the flow’s rate of the perfusate, the fluid viscosity, or the channel’s height and width [179].

The parallel flow technique was first introduced to study endothelial cell adhesion [178,179] and has been further explored in the adhesion studies of biotinylated endothelial cells adhered on glass with fibronectin or RGD peptide functionalization [180,181,182,183]. Tapered height chamber have been developed to produce linear variations of shear stresses along channels at a single flow rate [184]. Cao *et al.* [185] modified the chamber to developed a side-view chamber system where side-view images of cellular deformation and adhesion to various adhesive surfaces under dynamic flow conditions could be observed. Cellular adhesion to surfaces functionalized with artificial ECM proteins and polymer surfaces treated by plasma using different gaseous substances have also been studied [186,187]. The parallel flow technique has been been used to investigate the adhesion potential of various cell types to a range of different materials, including poly-l-lactide (PLL) films [187,188,189], polyelectrolyte multilayer films [190], polyethylene films [191], and numerous glass-treated surfaces [177,178,192,193,194]. Interestingly, the flow chamber can be used to observed cells’ vascular adhesion potential to the endothelial monolayer, which represents the human endothelial vein system. Gerszten *et al.* [176] were able to study the adhesion of monocytes to vascular cell adhesion molecule-1 (*VCAM-1*) transduced human endothelial cells under physiological flow conditions. Palange *et al.* [195] investigated the extravasation ability of circulating tumor cells (CTCs) to the endotheial cell layer under flow and the potential of natural compounds and curcumin treatment to attenuate the cell’s metastasis potential.

**Microfluidics**. Microfluidic lab-on-a-chip technologies represent a revolution of the flow chamber in laboratory experimentation, bringing the benefit of miniaturization, integration, and automation to many research-based industries. These greatly reduce the size of the devices and make many portable instruments affordable with quick data read-outs. The use of small sample volumes leads to greater efficiency of chemical reagents, straightforward construction and operation processes, and low production costs per device, thereby allowing for disposability and fast sampling times. The ability for real-time observation makes microfluidics bring high promises for cell adhesion studies. In recent years, cell adhesion studies have been carried out in a miniature form of the traditional parallel plate flow chambers as discussed above, using flow in rectangular microchannels to apply shear stresses to cells. These devices are typically constructed from optically transparent PDMS bonded to glass using the soft lithography rapid prototyping process that allows many nearly identical devices to be manufactured in a short amount of time [196]. The optically transparent criteria is important to enable the use of different real-time microscopy techniques to explore cell behaviors under diverse experimental conditions [197]. Small dimensions associated with micrometer-sized channels ensure laminar flow even at very high linear fluid velocities, which is often required when large shear stresses are generated [198].

A microfluidic device consisting of eight parallel channels has been used to assess the effect of varying collagen and fibronectin concentrations on the adhesion strength of endothelial cells [197]. A series of microfluidic channels have been constructed to investigate the adhesion strength of fibroblast cells adhered to fibronectin-coated glass surfaces [198]. In another case, microchannel assays were used to examine the adhesion of various cell types on surfaces with various coatings, including collagen, glutaraldehyde, and silane [199]. Kwon *et al.* [19] used a microfluidic shear device consisting of four parallel channels with different surface topography patterns to separate cancer cells mixed in a population of healthy cells based on adhesion strength. A shear stress-dependent cell detachment from a temperature-responsive cell culture using a microfluidic device has been developed to quantitatively estimate the interaction between cells (*NIH/3T3* mouse fibroblast and bovine aeortic endothelial cells) and materials [200]. Recently, microfluidic technology has moved forward to the studies of single cells. Honarmandi *et al.* [24] integrated microfluidics with optical tweezers for the study of mechanotransduction and focal adhesion of single endothelial cells. Microfluidic devices were `used to provide convenient means of positioning a cell into a specific location in the channel with controlled physiological conditions while the optical tweezers were used to detach the adhered cells. Christ *et al.* [107] have upgraded the application of microfluidics from the study of population cell adhesion to single cell studies. A rectangular microchannel was used to analyze the adhesion strength of single *NIH3T3* fibroblast cells that had been allowed to adhere for 24 h on the collagen and fibronectin coatings on glass [107]. In this work, single cells were imaged throughout the detachment process, and the relationship between adhesion strength and cell geometry was investigated.

## 3. Advantages and Limitations of the Techniques Used in Cell Adhesion Studies

Single cell approaches allow for precise measurements of the separation of the cell from the substrate. Specialized equipment, which is bulky and expensive, is often required for manipulation and alignment of the probe and testing can be time-intensive. The single cell adhesion measurement approach provides more precise measurement of the individual cell when compared to the population cell approach. The single cell measurement approach allows the system to image biomolecules at nanometer-scale resolution, to have a dynamic range of forces able to be applied to cells, and to process samples in their physiological medium and aqueous buffer [120]. It has been widely used in the cells’ teether (adhesion process) formation [126] and in the rupture forces [125,128,201] of the molecular adhesion bonds that couldn’t be measured by most of the cell adhesion strength measurement methods. However, beside the precision of the techniques, limitations such as low-throughput measurement, high equipment cost, time consumption, the need of a skilled operator, and other operator variables in the data obtained are unavoidable. These restrictions underscore the need for developing additional simple techniques that do not require expensive equipment, and are able to measure changes in cell adhesion properties associated with diseases or specific physiological perturbations. Some of the methods require computational processing and high-end confocal microscopes, which are not available in most laboratories. Table 2 summarizes the advantages and limitations of techniques used to study cell adhesion. The population cell approach provides data from the average response of a group of cells. Even though this approach could not provide precise data on the characteristic of an individual cell, the approach was still widely used to study cell adhesion until recently. The importance of the approach can not be denied as it provides essential information in the medical field for disease treatments, tissue engineering, and biomaterial compatibility.

**Table 2:** Comparison of advantages and limitations in the techniques used for cell adhesion studies.

Method	Strength	Weaknesses	References
Polyacylamide-traction Force Microscopy (PA-TFM)	Real time observation; No special and expensive equipment needed for fabrication Inexpensive; Flexible to chemical and mechanical adjustment; Adaptable to a large variety of cells	Needs to record both unstressed and stressed state of the substrate; Suffers from uncertainties in tracking beads’ position	[47,48,49,50,51,52,53,54]
Micropatterning (Micropost array/micropillar)	Real-time observation; Force quantification easier and more reliable than PA-TFM; The micropillar stiffness is manipulated by its geometry; Gives good precision over surface chemical properties on micrometer scale	Substrate can alter cell’s behavior; Requires sophisticated equipment to fabricate; Needs skilled operator; Sensitivity of the microposts to the particular cell type needs to be optimized	[26,55,56,57,58,59,60]
Three Dimensional Traction Force Quantification (3D-TFM)	Real-time observation; Flexible to chemical and mechanical adjustment; Adaptable to a large variety of cells; Flexible to chemical and mechanical adjustment; Adaptable to a large variety of cells	Needs high-end confocal microscope; Needs high computational processing; Needs to record both unstressed and stressed state of the substrate; Suffers from uncertainties in tracking beads position	[61,62,63,64,65,66,67,68,69,70]
Wash Assay	Simple	Not a quantitative data, needs further analysis to obtain quantitative data; Poor reproducibility; Insensitive	[71,72,73,74]
Resonance Frequency	Real-time observation; Real-time measurement	Poor reproducibility	[36,76,77,78,79,80,81,82,83,84,85,86,87,88,89,90,91,92,93,94,95]
Microfluidics	Straightforward construction and operation; Real-time observation and measurement; Convenience in size (compatible with cell sizes); Fast and simple to operate; Non-invasive to cell	Low detachment force; Restricted to short-term adhesion	*Attachment events* [96,97,98,99,100,101,102,103,107]; *Detachment Events* [19,24,107]; [197,198,199,200]
Cytodetachment	Real-time observation; Quick detachment of cell; Range of force produced is high and applicable to long-term adhesion	Alignment of probe and cell; Time-consuming; Needs highly skilled (experienced) operator; Operator variable; Cell damage (hard contact); Expensive equipment; Not real-time measurement	[104,105,106]
Micropipette Aspiration	Real-time observation and measurement; Common lab equipments	Alignment of probe and cell; High skilled (experienced) operator; Operator variable; Cell damage (hard contact)	[18,108,109]
SCFS-AFM probe	Real-time observation Precise data for short term adhesion studies	Alignment of probe and cell require micromanipulator; Time consuming; Need skilled operator; Operator variable; Cell damage (hard contact); Expensive equipments; Not real-time measurement	[105]; [116,117,118,119,120,121,122,123,124]
SCFS-Biomembrane Probe	Real-time observation; Precise data for short term adhesion studies	Low maximum force (pN); Restricted to short term adhesion; High skilled (experienced) operator; Operator variable; Probe variable (fluctuation of probe due to thermal excitation)	[125,126,127,128]
SCFS-Optical Tweezer	Real-time observation; Precise data for short term adhesion studies; Compatible with microfluidic device	Low maximum force (pN); Restricted to short term adhesion; High skilled (experienced) operator; Operator variable; Cell damage	[129,130,131,132,133,134,135,136,137,138]
Centrifugation	Many analysis can be examined in parallel; Common lab equipments	Low maximum force (uncomplete detachment); Only a single force can be applied per experiment; Nota real-time analysis	[139,140,141,142,143,144,145,146,147]
Spinning Disk	A range of stresses able to be applied in single experiment; High stresses	Not a real-time analysis; Custom-made apparatuses	[148,149,150,151,152,153,154,155,156,157,158,159,160]
Flow chamber: Radial flow; Parallel flow	Radial flow: Ranges of stresses applicable in single experiment; Real-time cell detachment observation; Paralel flow: Simple fabrication; Straightforward operation; Real-time cell detachment observation	Radial flow: Low detachment force; Restricted to short term adhesion; Paralel flow: Low detachment force; Restricted to short term adhesion	[163,164,165,166,167,168,169,170,171,172,173,174,175]; [176,177,178,179,180,181,182,183,184,185,186,187,188,189,190,191,192,193,194,195]

## 4. Summary

Studying human diseases from a biomechanical perspective can lead to a better understanding of the pathophysiology and pathogenesis of a variety of illnesses because changes occurring at the molecular level will affect, and can be correlated to, changes at the macroscopic level. Research on biomechanics at the cellular and molecular levels not only leads to a better elucidation of the mechanisms behind disease progression, it can also lead to new methods for early disease detection, thus providing important knowledge in the fight and treatments against the diseases. Sickle cell disease (SCD) could be characterized by observing the red blood cells’ (RBC) adhesiveness and deformability [98,202,203,204,205]. Cell adhesiveness was found to be reduced in human cancers. Diseased cells’ properties have been found to be physically different from that of healthy cells [206]. The adhesion strength of cancer cells was found to be lower than the normal cells [19] and decreased in line with their increased “metastatic potential” [18]. Reduced intercellular adhesiveness allows cancer cells to disobey the social order, resulting in the destruction of the histological structure, which is the morphological hallmark of malignant tumors [8]. Polymorphonuclear leukocytes (PMNs) migrate from the bloodstream to the inflammation sites by adhering to the surface of the endothelium during infection and tissue injury [207]. The knowledge obtained can also be useful in the development of new and improved assays and diagnostic devices, and the techniques are not only sensitive enough for the early detection of diseases, but they are also highly accurate, so it is possible to detect diseases when the symptoms or signs are hardly discernable. Determining chronic diseases in their initial stages is promising in curing the illness and saving lives, thus improving the quality of human health.

**Figure 6:**
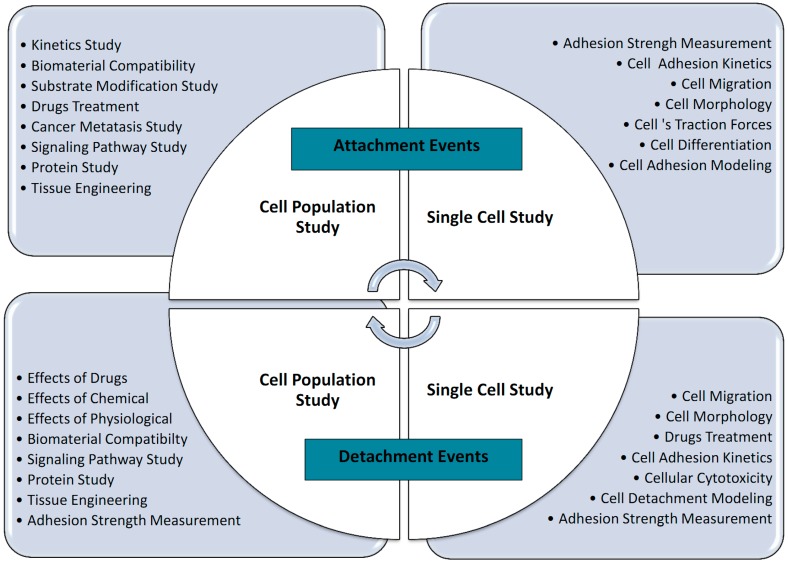
Summary of the importance of adhesion studies and their applications.

Figure 6 summarizes the importance of cell adhesion studies and its applications categorized by attachment and detachment events and grouped by single cell and population studies. Cell adhesion studies cover a wide range of important applications from the fundamental single cell adhesion behavior (morphology, migration, kinetics) and understanding the cell signaling pathway to how the physiological factors (temperature, pH, fluid flow), treatments (chemical, drugs, toxic, different substrate) and conditions affect cell adhesion and cancer metastasis studies as well as tissue engineering and biocompatibility studies for implants. This essential information obtained from the adhesion studies leads to the development of the computational model for further studying and understanding cell adhesion [27,96,208,209,210,211,212,213,214,215,216,217]. The future potential of single cell adhesion characterization is especially significant for early disease diagnosis. This emerging field can lead to the development of biomarkers for chronic diseases and cancers in their early stage at the cellular level. Furthermore, the new techniques or devices will bring high promises in the search of suitable treatments for those who have diseases in their early stages. Beside the importance of single cell adhesion, the cell adhesion population approach plays an important role in and brings high promises for the development of biomaterials in tissue engineering for implantable bioMEMs/biosensors, tissue scaffold production, and the applications of artificial bones as well as tooth replacement. Cell adhesion population studies are also essential in analyzing the potential of drug treatments, improving drug delivery systems, attenuating cancer metastasis development, understanding the dynamic mechanism of cell adhesion in many important biological processes, and finding a cure for many diseases or human health-realated problems.

## 5. Conclusions and Future Directions

Cell adhesion is a very important process in the human biological system. Studying both cell attachment and detachment events provides essential knowledge in understanding many important functional processes in the human body, which lead us to find the causes and problems that trigger certain diseases and thus develop the strategy for curing and improving them. Many different techniques and adhesion assays have been developed to study cell adhesion applicable to a wide range of fields. Every method is unique and was developed for specific important and independent purposes, which makes them difficult to compare in finding the best method applicable for cell adhesion studies. Choosing an appropriate technique is highly dependent on the purpose of the information that a person desires to obtain. Both single cell and population studies are equally important and required to fully understand how cells behave and function in the human system.
